# Détention provisoire des jeunes femmes accusées d'avortement clandestin ou d'infanticide au Sénégal

**Published:** 2012-06-26

**Authors:** Mohamed Maniboliot Soumah, Liliane Flore Pemba

**Affiliations:** 1 Service de Médecine Légale, Faculté de Médecine, Pharmacie et Odontostomatologie, Université Cheikh Anta Diop (UCAD), BP 7080, Dakar, Sénégal

**Keywords:** Infanticide, avortement, détention provisoire, Sénégal

## Abstract

**Introduction:**

L'activité sexuelle chez les jeunes les expose à un accroissement du risque de contracter des grossesses non désirées. Le recours à l'avortement clandestin avec son corollaire de complications peut entrainer le décès de la jeune femme. Avortement et infanticide sont interdits et sanctionnés par la loi sénégalaise. Comment ces jeunes femmes vivent-elles leur détention? Existe-il des alternatives à la détention pour éviter leur désocialisation?

**Méthodes:**

Cette étude rétrospective portait sur la maison d'arrêt des femmes de Dakar située à Liberté 6, un quartier de Dakar. Nous avons procédé à des entretiens avec des femmes détenues à la maison d'arrêt des femmes de Dakar et suspectées d'infanticide ou d'avortement clandestin.

**Résultats:**

Les femmes de notre échantillon ont une moyenne d’âge inférieure à 25 ans avec parmi elles une fille mineure de 16 ans. Nous avons trouvé 18,51% de femmes suspectées d'infanticide ou d'avortement. Dans notre étude 50% des femmes sont originaires de la périphérie et de la banlieue de Dakar et presque 44% proviennent des autres régions du pays. La durée moyenne de détention provisoire est de neuf mois.

**Conclusion:**

Malgré leur qualification distincte dans le code pénal: l'infanticide est un crime et l'avortement un délit, les femmes suspectées d'avoir commis ces actes sont soumises à de longues détentions préventives.

## Introduction

La pratique de la sexualité avant le mariage est observée dans la plupart des pays africains, en raison du recul de l’âge du mariage et de la précocité des rapports sexuels. Cette activité sexuelle chez les jeunes, les expose au risque de contracter des grossesses non désirées [1, 2]. Leurs conséquences sont l'exclusion scolaire et sociale. L'interruption volontaire de grossesse n'est pas permise par la loi. En cas de grossesse non désirée, le recours à l'avortement clandestin parait la seule alternative, avec son corollaire de complications. Quand la femme survit à l'avortement, elle risque l'emprisonnement car l'avortement est un acte illégal réprimé par la loi. Lorsqu'elle n'a pas eu recours à l'avortement, la jeune mère célibataire sera suspectée d'infanticide si le nouveau-né décède dans les jours ou semaines après l'accouchement.

Ce travail vise à préciser les caractéristiques épidémiologiques, sociologiques et médico-légales des femmes auteurs d'infanticide ou d'avortement afin d'améliorer la législation en la matière. Comment les jeunes femmes vivent-elles leur détention? Existe-t-il des alternatives à la détention afin de limiter leur désocialisation?

## Méthodes

L’étude rétrospective portait sur la maison d'arrêt des femmes de Dakar située au quartier Liberté 6. Nous avons procédé à des entretiens avec des femmes détenues à la maison d'arrêt des femmes de Dakar et suspectées d'infanticide ou d'avortement clandestin, après autorisation de l'administration pénitentiaire. Chaque détenue était libre d'accepter ou non l'entretien. Ont été inclues toutes les femmes accusées d'infanticide ou d'avortement ayant accepté de participer à l'enquête. Les entretiens se sont déroulés du 9 juin au 12 juillet 2006 lors d'un stage de médecine pénitentiaire autorisé par l'administration pénitentiaire. Les détenues étaient vues séparément par un enquêteur unique, en présence de l'infirmière. Pendant cette période, il y avait 108 détenues dont 20 femmes étaient suspectées d'infanticide ou d'avortement. Parmi celles-ci, 16 ont accepté de répondre à nos questions. Les critères recherchés étaient l’état civil des femmes interrogées (identité, statuts professionnel et matrimonial), le type sociologique de famille dont elles sont issues, leurs connaissances en matière de sexualité, le déroulement de la grossesse et le vécu de l'incarcération.

## Résultats

La maison d'arrêt des femmes a été créée en 1992. C'est un établissement qui peut recevoir jusqu’à 100 détenues. Ces détenues se répartissent de la façon suivante: les détenues provisoires, les personnes soumises à la contrainte par corps, les personnes condamnées à des peines inférieures à un an, les personnes ayant interjeté un appel de leur condamnation.

### Age

L’âge moyen des détenues vues au cours de l'enquête est de 23,93 ans. La plus jeune était âgée de 16 ans et la plus âgée avait 29 ans. Dix détenues avaient entre 21 et 25 ans, 4 d'entre elles avaient entre 26 et 30 ans et 2 détenues avaient entre 16 et 20 ans.

### Structuration de la famille originelle

Dix femmes étaient issues de familles polygames soit 62,5% de notre échantillon. Dans ces familles polygames, le nombre moyen d’épouses était de 2,4 épouses avec des extrêmes allant de 2 à 4 épouses par foyer.

Concernant le rang des femmes interrogées dans leur fratrie, nous n'avons tenu compte que de la fratrie utérine. Parmi celles-ci, 6 femmes (37,5%) étaient des aînées et 3 (18,75%) étaient des benjamines. La taille moyenne de ces fratries utérines était de 5,9 enfants avec des extrêmes allant de 3 à 10 enfants.

### Type de famille

Dans notre enquête la majorité des femmes interrogées est issue d'une famille sociologiquement adéquate (11 femmes soient 68,75%) et accessoirement de famille malhabile (4 femmes soient 25%) et conflictuelle (1 femme soit 6,25%). Nous n'avons pas trouvé de femmes issues de familles déviante ou punitive.

### Origine géographique

Neuf femmes (56,25%) sont originaires de Dakar et parmi elles une seule est originaire de Dakar centre; les huit autres sont originaires de Dakar périphérie et Dakar banlieue avec respectivement quatre femmes pour chaque localité. Sept femmes (43,75%) sont originaires d'autres régions du pays.

### Niveau de scolarisation

Les femmes de notre échantillon sont caractérisées par un faible niveau de scolarisation. Une seule avait atteint le cycle secondaire. Cinq détenues avaient reçu une instruction coranique et 10 autres avaient arrêté leurs études au niveau primaire.

### Activité professionnelle

Parmi les femmes exerçant une activité, une cumulait une activité salariée et une activité informelle. Six femmes étaient salariées, 4 femmes travaillaient dans le secteur informel et 7 femmes n'avaient aucune activité génératrice de revenus. Onze femmes bénéficiaient de l'assistance d'une autre autorité économique.

### Statut matrimonial

La série comptait neuf femmes célibataires, deux femmes mariées et cinq femmes divorcées. Parmi les neuf femmes célibataires, quatre avaient déjà un enfant; quant à celles qui sont mariées, l'une d'entre elles vivait seule du fait de l'immigration de son époux.

### Informations à propos de la sexualité

Douze femmes (75%) nous ont déclaré avoir été informées sur la sexualité. Leurs sources d'informations étaient essentiellement constituées par les médias et l'entourage; une seule avait bénéficié de cours d’éducation sexuelle pendant sa scolarisation. La famille directe n'a pas été citée comme source d'informations. Si plus de la moitié des femmes (62,5%) ont dit avoir été informées des moyens de contraception, seules 18,75% ont déclaré les avoir utilisés. Les sources d'information concernant la contraception étaient les mêmes que pour l'information sur la sexualité.

Dans la majorité des cas (81,25%), le rapport sexuel à l'origine de la grossesse était consentant; il a été fait par contrainte dans trois cas. Aucune femme n'a déclaré avoir eu des rapports sexuels pour des besoins financiers ni se livrer à la prostitution.

### Informations concernant la grossesse

Parmi les femmes de notre échantillon, la moitié avait fait des consultations prénatales avec une moyenne de trois consultations prénatales et des extrêmes allant de une à six consultations prénatales.

L'entourage et la famille de la moitié des femmes interrogées étaient informés de l’état de grossesse. Si le partenaire était connu dans tous les cas, il avait été informé de l’état de grossesse dans dix cas, il était alors prêt à assumer ses responsabilités La grossesse s'est terminée par un avortement dans trois cas dont un avec l'aide d'un agent de santé. Pour celles qui ont accouché, quatre ont eu un accouchement prématuré, cinq un accouchement à terme et pour les quatre autres le terme n’était pas connu. Soit un total de 3 avortements clandestins et 13 infanticides.

Concernant les conditions de l'accouchement, seules deux femmes ont accouché dans un centre de santé; une a accouché à domicile avec l'assistance d'une parente, les dix autres étaient seules au moment de l'accouchement à domicile.

### Statut du fætus

Presque toutes les femmes interrogées (81,25%) ont déclaré avoir pris conscience dès le début de la grossesse que l'enfant porté était un être humain; seules deux (12,5%) l'ont réalisé à l'accouchement. Treize femmes (81,25%) ont déclaré qu'elles auraient voulu garder leur enfant mais en changeant de village pour l'une d'entre elles. Une femme voulait faire adopter son enfant.

### Visites lors de la détention

Toutes ces femmes reçoivent des visites de leurs proches. Ces visites sont au moins hebdomadaires pour 68,75% des femmes et mensuelles pour 25% d'entre elles. Une seule reçoit des visites très rarement du fait de l’éloignement de sa famille par rapport à la maison d'arrêt. La durée moyenne de leur incarcération au moment de notre enquête était de 8,9 mois avec des extrêmes allant de 3 à 22 mois.

Toutes les femmes bénéficient d'un examen médical à l'admission en prison. Au cours de cet examen sont notés les renseignements concernant la date des dernières règles et les plaintes éventuelles. Au cours de notre enquête près de la moitié des femmes de notre échantillon (43,75%) nous a déclaré entretenir avec les autres détenues des rapports d'amitié; les autres manifestaient plutôt de l'indifférence à l’égard de leurs codétenues.

## Discussion

Les femmes de notre échantillon ont une moyenne d’âge inférieure à 25 ans avec parmi elles une mineure de 16 ans. La jeunesse de la population carcérale féminine est un caractère qui est retrouvé dans la littérature notamment au Mexique et en Croatie [3–6]. Elles sont issues pour la plupart de familles polygames et nombreuses. La grande majorité d'entre elles nous ont déclaré appartenir à des familles adéquates dans lesquelles, selon les sociologues, l'activité délictueuse est occasionnelle. Mais le grand nombre d'individus au sein des familles (polygamie et importante fratrie) entraine une faible supervision par le chef de famille.

Au cours de notre enquête, nous avons trouvé 18,51% de femmes suspectées d'infanticide ou d'avortement. Dans sa thèse faite en 1995, SENE avait relevé que 20% de femmes étaient incarcérées pour infanticide [3]. Ces chiffres sont certainement sous estimés du fait de l'illégalité de l'acte, seules les femmes qui font l'objet d'une dénonciation se retrouvent en prison [7].

Le lieu de résidence et le faible niveau de scolarisation des détenues avaient également été notés par SENE [3, 8]. Dans notre étude 50% des femmes sont originaires de la périphérie et de la banlieue de Dakar et presque 44% proviennent des autres régions du pays. C'est la même chose pour les autres détenues; les zones périphériques et la banlieue sont plus peuplées et plus pauvres avec une criminalité plus importante. La grande majorité a quitté l’école avant même la fin du cycle primaire. Elles survivent en exerçant des activités qui requièrent peu ou pas de qualifications notamment les travaux domestiques pour celles qui sont salariées et le petit commerce pour celles exerçant une activité informelle. Quelques femmes associent ces deux types d'activité afin d'avoir des moyens substantiels pour une meilleure qualité de vie. C'est cette quête de meilleures conditions de vie qui est à l'origine de l'exode des autres régions du pays vers la capitale.

Les femmes étaient seules du fait d'un célibat ou d'un divorce ou encore du fait de l’éloignement de l’époux pour des raisons économiques. Malgré cette solitude, plus de la moitié d'entre elles, avait informé l'entourage proche de leur état de grossesse. Presque toutes ces femmes auraient voulu garder leur enfant et le partenaire, quand il avait été prévenu, était prêt à assumer ses responsabilités vis-à-vis de l'enfant à naître. Mais on constate que trois quarts des femmes interrogées étaient seules au moment de l'accouchement ou de l'avortement; même celles qui avaient fait des consultations prénatales avaient accouché à leur domicile. Ceci peut s'expliquer par le sentiment de honte et de culpabilité que peuvent éprouver ces femmes dans une société o[ugrave] la maternité ne se conçoit que dans le cadre du mariage. Dans une étude, CARR et col avaient noté que moins de 55% de jeunes femmes déclaraient avoir reçu des soins de délivrance professionnels [9]. Les contraintes sociales semblent les pousser à commettre l'irréparable, acte qui les marginalise un peu plus quand il est découvert du fait du caractère sacré de la vie. Le Sénégal est un Etat de l'Afrique de l'Ouest d'une superficie de 196 722 km2 avec une population de 11113000 habitants dont 2555990 habitants à Dakar, la capitale soit 23%. Le budget de la santé est de 70,5 milliards de francs CFA correspondant à 10% du budget national. L'indice synthétique de fécondité est de 5,3, le taux de prévalence contraceptive est de 10,3% pour la population générale. Ce taux baisse à 5% pour la tranche d’âge 15–19 ans et 8,4% pour la tranche d’âge 20–24 ans. Le taux d'accouchements assistés par un personnel de santé qualifié est de 51,9%. Le taux de mortalité maternelle est de 434 décès pour 100 000 naissances vivantes (Source ministère de la santé 2007). Les contraceptifs sont disponibles et subventionnées dans les structures de santé publiques. Les contraceptifs existent aussi dans les officines privées.

La qualité des informations à propos de la sexualité et des moyens de contraception reçues par ces femmes est peu fiable du fait de leurs sources essentiellement constituées des médias et de l'entourage. Ces sujets ne sont pas abordés par les parents qui les considèrent comme tabou en Afrique. Selon GUEYE et col, la modernisation et l'exposition croissante aux médias parallèles, le déclin de l'autorité des parents et des aînés ont amoindri les règles sociales et culturelles qui régissaient et informaient auparavant la sexualité des adolescents. Face à l'intensification des exigences matérielles, les besoins économiques des adolescentes sont devenus une responsabilité individuelle et non plus familiale. Les principales sources de revenu des jeunes citadines sont les hommes plus âgés, disposant probablement de meilleures perspectives matérielles que les jeunes hommes de leur âge [10].

Les détentions provisoires prolongées sont une réalité fréquente dans les prisons africaines [11], cela est retrouvé dans notre étude. Dans notre étude la durée moyenne de détention provisoire avoisine neuf mois. Cette détention provisoire au plan national était d'un jour à 6 mois pour 62,5% des détenus provisoires, de 6 mois à 2 ans pour 24,85% et de 2 à 4 ans pour 8,8% (source Direction de l'Administration Pénitentiaire 2005). En matière correctionnelle les conditions de mise en détention provisoire d'un inculpé ont été rendues plus difficiles (réformes successives de l'article 127 du code de procédure pénale par les lois 85–25 et 99-06 du 13 janvier 1999); la durée de validité du mandat de dépôt délivré en matière correctionnelle qui était fixée à six mois renouvelables (article 127 bis loi 85-25) n'est plus renouvelable (même article loi 99-06) et le contrôle judiciaire a été introduit en 1985 comme palliatif à la détention provisoire. Mais en matière criminelle il n'y a pas de limitation de la détention préventive. Malgré cette longue détention, les femmes ne sont pas pour autant abandonnées par leurs familles.

Le contexte socio-économique ne semble pas être le seul facteur ayant conduit à la réalisation de l'infraction, d'autres facteurs psychologiques interviennent également [12, 13]. Au moment de l'accouchement, elle agit sans aide et sans présence d'un tiers. Cette peur qui s'est installée en elle depuis la découverte de son état détruit l’équilibre des valeurs; seule la mort du nouveau-né lui paraît alors moins grave que la désapprobation sociale et le scandale.

Des antécédents de traumatisme psychologique peuvent aussi constituer un facteur de risque d'infanticide [14]. Le sentiment de honte consécutif à des abus sexuels peut se convertir en un acte violent à la faveur d'une situation qui associe réminiscence et vulnérabilité [15]. Dans notre étude la notion d'abus ou d'agressions sexuels a été retrouvé dans près de 19% des cas (3 cas sur 16).

L'avortement clandestin et l'infanticide sont surtout dus au sous développement. Ils constituent un continuum pour la femme qui a une grossesse non désirée en Afrique. Si elle ne réussit pas l'avortement en début de grossesse, elle pratiquera un infanticide à l'accouchement. L'avortement thérapeutique n'est prévu que lorsque la vie de la mère est menacée par la poursuite de la grossesse. Les malformations du fætus n'autorisent pas l'interruption de la grossesse dans notre loi pénale et dans le code de déontologie médicale.

La pauvreté, le bas niveau d'instruction scolaire et l'ignorance des moyens contraceptifs concourent à la survenue de grossesses non désirées. La loi n'offre aucune solution car nos lois sont fortement inspirées de notre culture, notre tradition et nos religions. L'adoption n'est pas courante dans nos pays et les seuls centres de recueil pour les enfants sont en général des æuvres confessionnelles.

L'accent doit être mis sur la prévention: en évitant les grossesses non désirées par une meilleure information et une vulgarisation des méthodes contraceptives; en assouplissant la loi criminelle, permettant l'interruption volontaire de grossesse dans certaines conditions notamment en cas de viol; en durcissant la répression pour les auteurs d'agression sexuelle; en améliorant les conditions de vie des populations, en somme sortir du sous développement; en réduisant les durées de détention provisoire en matière criminelle; en faisant la promotion de l'adoption.

## Conclusion

La pratique de l'avortement clandestin et d'infanticide chez des jeunes mères seules n'est pas négligeable; celles qui se retrouvent à la maison d'arrêt des femmes ne représentent que la partie visible de l'iceberg. Les femmes suspectées d'avoir commis ces actes sont à priori soumises à de longues détentions préventives. Ces femmes pourraient bénéficier de la réforme relative aux procédures d'exécution et d'aménagement des sanctions pénales, afin de leur éviter de longues détentions sources de désocialisation. La quasi-inexistence de structures d'aide et d'accompagnement de jeunes mères célibataires ainsi que la pratique peu développée de l'adoption doivent inciter à la promotion de l’éducation des jeunes en matière de sexualité.
